# Eosinophilic Asthma, Phenotypes-Endotypes and Current Biomarkers of Choice

**DOI:** 10.3390/jpm12071093

**Published:** 2022-06-30

**Authors:** Konstantinos Porpodis, Ioanna Tsiouprou, Apostolos Apostolopoulos, Polyxeni Ntontsi, Evangelia Fouka, Despoina Papakosta, Harissios Vliagoftis, Kalliopi Domvri

**Affiliations:** 1 Pulmonary Department, Aristotle University of Thessaloniki, George Papanikolaou Hospital, 57010 Thessaloniki, Greece; kporpodis@yahoo.gr (K.P.); joanna_tsi@hotmail.com (I.T.); aposapos92@gmail.com (A.A.); evafouka@gmail.com (E.F.); depapako@gmail.com (D.P.); 2 2nd University Department of Respiratory Medicine, Attikon Hospital, 12462 Athens, Greece; xenia-1990@hotmail.com; 3 Department of Medicine, University of Alberta, 567 HMRC, Edmonton, AB T6G 2S2, Canada; hari@ualberta.ca; 4 Laboratory of Histology-Embryology, Medical School, Aristotle University of Thessaloniki, 54124 Thessaloniki, Greece

**Keywords:** asthma, eosinophilic, biomarkers, phenotypes, endotypes, biologic agents

## Abstract

Asthma phenotyping and endotyping are constantly evolving. Currently, several biologic agents have been developed towards a personalized approach to asthma management. This review will focus on different eosinophilic phenotypes and Th2-associated endotypes with eosinophilic inflammation. Additionally, airway remodeling is analyzed as a key feature of asthmatic eosinophilic endotypes. In addition, evidence of biomarkers is examined with a predictive value to identify patients with severe, uncontrolled asthma who may benefit from new treatment options. Finally, there will be a discussion on the results from clinical trials regarding severe eosinophilic asthma and how the inhibition of the eosinophilic pathway by targeted treatments has led to the reduction of recurrent exacerbations.

## 1. Introduction

The Global Initiative for Asthma (GINA) defines asthma as “a heterogeneous disease, usually characterized by chronic airway inflammation with a severe global impact on quality of life, mortality, economy, and health care utilization” [1,2]. Although asthma affects 1–18% of the population, its diagnosis remains a challenge in everyday clinical practice [3], leading to both over- and under-diagnosis, particularly in the elderly and in low- and middle-income countries [3,4,5]. Nowadays, the diagnostic algorithm is mainly based on GINA recommendations and differs in cases of patients already receiving controller treatment [6]. The clinical course of the disease presents inter-individual variability, suggesting distinct underlying pathophysiological mechanisms mediating symptoms and signs of the disease. These multiple pathophysiological mechanisms may also explain, at least to some extent, the differential response to therapy [7,8,9].

As a result, personalized approaches and treatments are valuable in the management of severe uncontrolled asthma. Severe asthma is nowadays described as “asthma that is uncontrolled despite adherence with maximal optimized high dose ICS-LABA and treatment of contributory factors, or asthma that worsens when high dose treatment is decreased”. Patients diagnosed with severe asthma experience a heavy burden of symptoms, exacerbations, and financial distress [5]. Despite the progress in pharmacological therapy and the continuously updating recommendations for asthma management, there is still an unmet clinical need to detect and treat the appropriate patients suffering from severe, uncontrolled asthma with new agents [10]. For this purpose, the identification of different phenotypes of asthma and biomarkers in everyday clinical practice to guide decisions is crucial.

This review discusses the distinct phenotype of eosinophilic asthma and the endotypes associated with eosinophilic inflammation. It also focuses on biomarkers that are used in everyday clinical practice and evaluates the use of eosinophilic-targeted treatment based on the results of several studies, aiming to inform clinicians on how to use clinical phenotypes to achieve an optimal personalized approach and further management.

## 2. Asthma Classification

The classification of asthma was initially developed on the role of allergens, and asthma was divided into extrinsic and intrinsic [11]. Classification systems were later based on clinical features (persistent airflow limitation or exacerbation-prone asthma), airway inflammation (eosinophilic, neutrophilic, mixed, or paucigranulocytic), and cluster analysis [12]. Specifically, according to GINA, “asthma phenotypes” are defined by recognizable clusters of demographic, clinical, and/or pathophysiological features [5,7], while the term endotype describes a subtype of a disease defined functionally and pathologically by a molecular mechanism or by treatment response [12,13]. Consequently, the PRACTALL consensus report in 2011 recommended the use of endotypes for the classification of asthma, since it could offer the possibility to optimize management and precision therapy [7].

In the context of classification strategies, Wenzel attempted to link biology to phenotypes and described six different categories in 2012, including early-onset allergic Th2, late-onset eosinophilic, exercise-induced, obesity-related, neutrophilic, and paucigranulocytic asthma [14]. Furthermore, Wenzel’s proposal to distinguish two subtypes of corticosteroid-dependent asthma depending on the level of bronchial eosinophilia led to the identification of two asthma endotypes: Th2-high and Th2-low [15]. The Th2-high endotype is usually associated with some degree of eosinophilic airway inflammation and a variable allergic or nonallergic background [16].

Overall, studies show that the most common and well-understood phenotype is eosinophilic asthma, as it affects over half of the patients that are diagnosed with severe asthma [17]. The diagnosis of eosinophilic asthma is based on the detection of sputum or peripheral blood eosinophilia and airway eosinophilic infiltration [18]. Numerous studies focus on the high importance of identifying different severe asthma phenotypes. Cellular phenotyping based on the type of airway inflammation is nowadays the most appropriate approach to guide the type of treatment the patient may benefit from in severe or difficult-to-treat asthma [12].

## 3. Eosinophilic Asthma

All subtypes of asthma were initially considered to be eosinophilic; however, over time, a thorough understanding of its pathogenesis led to the recognition of phenotypes associated with the underlying type of inflammation [11,19]. The complex role of eosinophils in the pathogenesis of asthma has been extensively investigated in the past two decades. These major effector cells mainly induce airway hyperresponsiveness and Th2 inflammation by releasing multiple molecules including cytokines (IL-2, IL-4, IL-5, IL-10, TNFα, TGFβ, etc.), chemokines (macrophage inflammatory protein 1 alpha, etc.), and granule proteins in response to allergens and parasitic, bacterial, fungal, and viral infection [20].

More specifically, eosinophilic asthma makes up approximately 70% of all severe asthmatic cases. It is characterized by tissue and sputum eosinophilia, the thickening of the basement membrane, and, usually, corticosteroid responsiveness [8]. Although a standard definition has not been developed yet, peripheral blood eosinophil counts of ≥150 cells/μL, ≥300 cells/μL, or ≥400 cells/μL have been used in trials to describe eosinophilic asthma and can readily be identified in a primary care setting [21,22]. There needs to be a more balanced discussion of the utility of conducting a blood eosinophil count in the primary care setting, as phenotyping/endotyping mainly applies for more severe cases under specialist care.

Exhaled nitric oxide (FeNO) levels are also used to suggest airway eosinophilia [21,23]. Experts of the ISAR Steering Committee developed a multicomponent, eosinophilic gradient algorithm based on variables including details on asthma onset, atopy, comorbidities, and biomarker concentration, aiming to facilitate asthma phenotyping and, ultimately, the selection of appropriate phenotype-specific treatment [24].

Eosinophilic asthma could be either atopic or non-atopic. Interestingly, patients with eosinophilic asthma could be further categorized into subtypes that may predict their response to specific therapeutic approaches. Unbiased clustering analyses resulted in the identification of different phenotypes associated with eosinophilic inflammation [16]. These phenotypes are: (1) childhood-onset atopic asthma, (2) adult late-onset eosinophilic asthma, and (3) aspirin-exacerbated respiratory disease (AERD).

### 3.1. Childhood-Onset Atopic Asthma

This phenotype is mainly characterized by the presence of clinically significant atopy/allergy, with Th2 inflammation as an underlying mechanism. The onset of symptoms dates to childhood and a diagnosis of eczema or another allergic/atopic condition in a patient predisposed to an allergic immune response. These children, due to their genetic and environmental background, could precede an asthma diagnosis [6,16]. In a patient predisposed to allergic immune response, the inhalation of aeroallergens initiates an inflammatory cascade including the differentiation of naïve T-cells into Th2-cells, the production of Th2-inflammatory cytokines, and IgE production by B-cells [18]. Airway and/or peripheral blood eosinophilia could be challenging to detect in the pediatric population. Eosinophilia is not uniformly present. However, studies have shown that airway and/or peripheral blood eosinophilia is attributed to IL-5 stimulation, resulting in the congregation of eosinophils.

### 3.2. Adult Late-Onset Eosinophilic Asthma

The initial description of this phenotype dates to 1947, when Rackeman detected a phenotype that was different from the classical childhood-onset allergic asthma [12]. Adult late-onset asthma presents in the fourth or fifth decade of life as a non-atopic and eosinophilic disease. Patients who are diagnosed with this phenotype often suffer from difficult-to-treat/severe asthma. A dominant feature is the presence of frequent exacerbations, poor control, persistent eosinophilic inflammation that may lead to a dependence on oral corticosteroids, and the early development of fixed airway obstruction and remodeling [6,16,25].

Several comorbidities are described, including other adult-onset eosinophilic airway diseases (allergic bronchopulmonary aspergillosis/mycosis (ABPA/ABPM), nonsteroidal anti-inflammatory drug-exacerbated airway disease (N-ERD), eosinophilic granulomatosis with polyangiitis (EGPA), etc.), which implies the potential existence of common genetic, immunological, and pathophysiological mechanisms among the aforementioned diseases [25].

Even though late-onset eosinophilic asthma resembles childhood-onset atopic asthma in Th2-high inflammation, there is no sign of elevated IgE. Persistent airway inflammation is caused by the production of IL-5 and IL-13 through allergen-independent ILC2s [12].

### 3.3. Aspirin-Exacerbated Respiratory Disease (AERD)

The prevalence of this phenotype ranges from 5.5 to 15% globally. The initial diagnosis usually takes place in the third decade of life, and it mainly affects women [12]. Asthma severity and prognosis vary among different cases. Patients experience severe and sometimes fatal exacerbations after the ingestion of aspirin or other NSAIDs [6,16]. This asthma subtype is usually linked to nasal polyposis and chronic sinusitis. The combination of aspirin sensitivity, asthma, and chronic rhinosinusitis with nasal polyposis forms the traditional description of “Samter’s Triad” [12].

Diagnosis can be challenging in the absence of “Samter’s Triad”, and an observed aspirin challenge may be required. Although atopy is usually related to AERD, evidence shows that the latter is not an allergic disease [6,16]. Specific asthma biomarkers are usually not useful in diagnosing AERD. Airway inflammation consists of elevated eosinophil levels along with the proliferation and increased activity of mast cells. COX-1 inhibition elevates the proinflammatory cysteinyl leukotrienes (LTC4, LTD4, LTE4) and reduces the level of anti-inflammatory PGE2 [6,16].

The successful treatment of AERD is based on aspirin desensitization, inhaler medications, and leukotriene modifiers. Current evidence suggests the use of anti-IgE treatment (omalizumab), as it has been shown to improve symptoms, quality of life, and lung function [12]. Recently, in a randomized crossover trial in AERD patients, it was demonstrated that subjective symptoms were improved with a reduction in nutritional salicylate intake [12]. Indeed, in addition to the therapeutic routines, a low dietary intake of food salicylate has been suggested in several studies as an adjunctive therapy for this condition [26].

## 4. Endotypes of Eosinophilic Inflammation

Current knowledge highlights the importance of moving from a clinical diagnosis of asthma followed by treatment to the identification of the specific asthma endotype for every patient followed by a patient-centered therapeutic approach based on the principles of personalized medicine. The history of endotyping dates to the mid-1920s; yet, there is no consensus on the definition of inflammatory endotypes—neither specific criteria nor a universal algorithm or classification system [7,11,27].

Since the landmark study that was conducted by Wenzel et al., and led to the recognition of two distinct inflammatory endotypes of severe asthma depending on the presence of eosinophils in endobronchial biopsy or lavage, research focuses on defining the right combination of different biomarkers to describe distinct endotypes [11,27]. Novel strategies are also used, such as omics-based technologies which are described in this review. Different endotypes could possibly co-exist in some patients. Currently, two endotypes based on biomarkers such as Th2 cells and type 2 cytokines are broadly used: Th2-high and Th2-low (or non-Th2) asthma [11,28].

The Th2-high endotype is the best-understood endotype. It is generally characterized by the presence of eosinophilic airway inflammation due to a Th2 cytokine response (IL-5, IL-4, IL-13, IL-25, IL-33) and thymic stromal lymphopoietin (TSLP). The IgE levels could be elevated but are not specific for any common antigen because of the lack of antigen presentation by antigen-presenting cells. The interaction between innate and adaptive immune responses results in Th2-high inflammation. The most important cells in this type are Th2 helper CD4+ cells, which lead to cytokine secretion and activate other innate and adaptive immune cells, basophils, mast cells, and B cells. In the airways, ILC2s generate Th2 inflammatory responses by producing IL-5 and IL-13. IL-5, IL-3, IL-4, IL-9, and IL-13 are the most important eosinophilic cytokines, and their function is to stimulate eosinophilic production, bone marrow extrusion, proliferation, and differentiation factors [11,19,29,30,31].

The current therapeutic approach for Th2-high severe asthma is based on biologics targeting allergy molecules (IgE) and eosinophilic interleukins (IL-5, IL-4, IL-13, TSLP). Due to the heterogeneity of Th2 endotype, the treatment response and clinical outcomes vary between patients. Many approaches have been proposed on how to select the appropriate agent; however, we still do not have a consensus on this issue [11,29].

The Th2-low endotype is characterized by excessive remodeling and a poor response to anti-inflammatory therapy. The underlying mechanisms in this asthma subtype are under investigation. Research suggests the existence of several modulators, such as age and metabolic or epigenetic factors, while the role of different pathways including IL-17, neutrophil intrinsic abnormalities, and the inflammasome pathway remains obscure [29,30]. Until now, there have not been useful biomarkers in clinical practice to predict T2-low asthma. MicroRNAs have recently drawn attention, and they are evaluated as potential biomarkers for T2-low asthma [11].

Recent progress in the treatment of severe asthma has been marked by the introduction of mixed endotypes. Patients could be classified into the Th1/Th2 and Th2 endotypes, which are associated with increased disease severity and a resistance to corticosteroids, or into TAC1, TAC2, and TAC3. The increased levels of IL1β, IL6, IL23, C3a, and serum amyloid A in patients with Th2/Th17-high type could enhance the development of patient-centered therapy [29].

## 5. Airway Remodeling/Smooth Muscle Function/Mucus Hypersecretion

Persistent airway inflammation aroused by eosinophils results in continuous tissue damage and airway remodeling, which is described by structural changes of the airways [32]. Airway remodeling is associated with fibrosis, angiogenesis, hypertrophy, and increased airway smooth muscle mass [33]. The combination of these results in airway wall thickening, luminal occlusion, and small airway obliteration. In addition, several inflammatory molecular factors are involved in these structural changes, such as platelet-derived growth factor (PDGF), transforming growth factor β (TGFβ), fibroblast growth factor (FGF), epidermal growth factor (EGF), TSLP, and cytokines that are produced in Th2- and non-Th2-inflammatory pathways, including IL-13, IL-4, IL-17, IL-21, IL-22, and TNFα [33].

Current evidence shows that airway remodeling may be a key feature of asthma endotypes, as eosinophils are associated with remodeling. Recently, the effect of the anti-interleukin 13 monoclonal antibody lebrikizumab in airway remodelling was investigated in a phase II bronchoscopy trial [34]. Researchers found that lebrikizumab treatment was associated with a reduced degree of subepithelial fibrosis apart from improved lung function and reduced key biomarkers in bronchial tissues. Moreover, according to research studies, the degree of remodeling depends on the severity of the disease, while its distribution is highly heterogeneous [35]. Although a close interaction with inflammation is established, causality is not yet clear. In fact, airway remodeling may occur in parallel with chronic inflammation or/and as a consequence of the inflammatory response [36]. On the other hand, the uncoupling of airway hyperresponsiveness and remodeling from airway inflammation has been recently described in T2-low asthma [37,38].

Concerning the role of bronchial smooth muscle in patients with asthma, it has been thoroughly researched in the past decades; however, the precise mechanism involved in its remodeling remains uncertain [35,39]. The smooth muscle increases airway inflammation by releasing numerous inflammatory mediators (e.g., endothelin, TGFβ), proliferating, and activating cells that are a part of different inflammatory pathways, such as T-lymphocytes [39]. The conduction of studies on novel therapeutics, including endothelin- or TGF-β-receptor antagonists could provide new data on personalized severe asthma therapy.

Furthermore, on the basis of bronchial smooth muscle, bronchial thermoplasty (BT) is an endoscopic method involved in the management of persistent, uncontrolled asthma, as it reduces airway smooth muscle mass (ASM) and nerve fibers in the airway epithelium [40]. Some studies demonstrated a clinical benefit after the use of BT, and that was depicted in improvements in lung function, asthma control, and quality of life and increases in symptom-free days [41,42]. On the other hand, the AIR2 trial raised concerns due to the high clinical meaningful improvement in the sham group, and in the randomized TASMA trial, the decrease in ASM mass failed to show a correlation with clinical outcomes [43,44,45]. Although the long-term follow up of the patients that participated in three randomized trials, AIR, AIR2, and RISA, showed sustained clinical improvement for ten years or longer, there is limited available evidence on the long-term safety and efficacy [42,44,46]. For these reasons, international guidelines do not recommend BT as a routine practice but only in the context of an independent Institutional Review Board-approved systematic registry or clinical study [5,47].

In addition, mucus hypersecretion has been related to asthma, as there is evidence of an increased MUC5AC presence in severe asthma epithelial cells [36]. Studies have shown that there is no association between the detection of mucus plugs and a differential response to therapy with the anti-IL-5Rα antibody benralizumab [39]. There is also evidence that mucus hyperplasia is promoted by the IL-4/IL-13 pathway. Therefore, biologic agents targeting this pathway, such as dupilumab, could be beneficial for patients presenting with mucus hypersecretion [48].

## 6. Current Trending in Eosinophilic Biomarkers

### Types of Biomarkers

**Figure 1:**
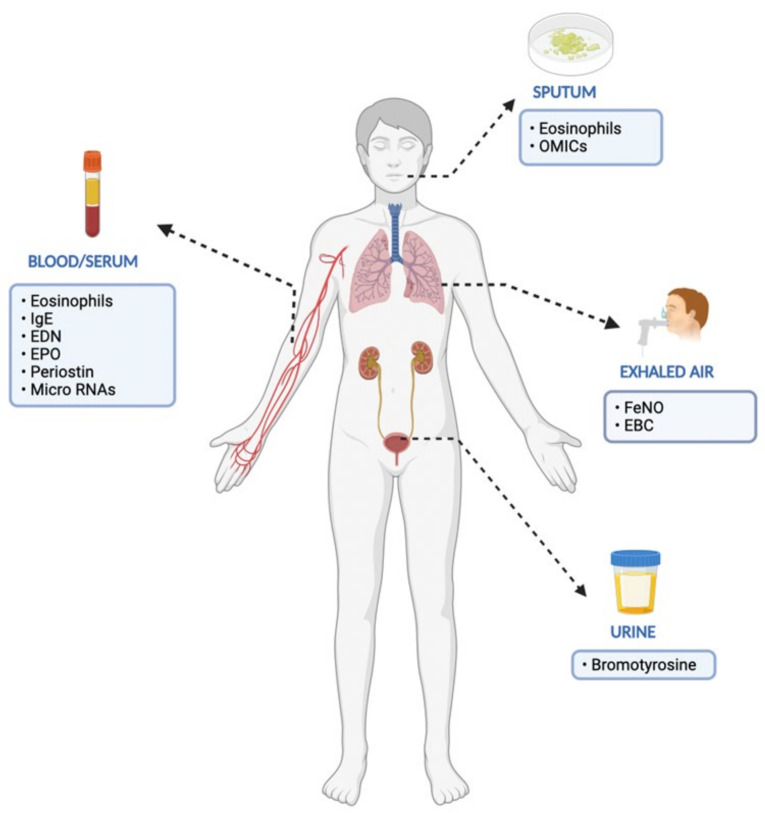
Established and proposed biomarkers in the management of severe eosinophlic asthma (EDN: eosinophil-derived neurotoxin, EPO: eosinophil peroxidase, EBC: exhaled breath condensate).

According to the 2016 FDA-NIH Biomarker Working Group, there are different types of biomarkers based on their main clinical application, as described in Table 1 [49]. Th-2 biomarkers such as urinary biomarkers and microRNAs or respiratory biomarkers have been revealed in several clinical trials (Table 2, Figure 1). Furthermore, ongoing research reveals that endotypes of asthma require more in-depth analysis and the use of omics technologies and systems biology [50].

## 7. Th2-High Biomarkers

### 7.1. Blood/Serum Biomarkers

Peripheral blood eosinophils are routinely counted in clinical practice and are often used as a surrogate of airway eosinophilia in severe asthma [51,52,53,54]. However, peripheral eosinophilia can also be found in other conditions such as parasitic infections and therefore lacks specificity [55]. Although blood eosinophilia has the highest accuracy among biomarkers in predicting sputum eosinophilia [54], there is also the possibility of a great discrepancy between blood and airway eosinophils, as the latter is more sensitive to predict Th2-high asthma [56]. It is noteworthy that 45% of patients with severe asthma will have a different cellular profile in induced sputum at one year [57].

Regarding severe asthma, persistent peripheral blood eosinophilia is associated with poor asthma control, followed by frequent exacerbations, hospital admissions, and gradual lung function decline [58,59]. Low blood eosinophil levels, similar to sputum eosinophils, are usually described in patients under therapy with anti-eosinophilic agents (mepolizumab) [60], reslizumab [61,62], benralizumab [63,64], tezepelumab [65], and corticosteroids [66]. This is why the role of blood eosinophils is currently discussed as a potential response biomarker for the above therapies. On the other hand, no significant change in eosinophil levels was observed after treatment with dupilumab [67].

IgE immunoglobulin is a principal molecule in allergic inflammation and contributes to the pathophysiology of severe asthma. Total IgE levels vary depending on numerous extrinsic and intrinsic factors, including IL-4 and -IL5 [68]. During severe exacerbations, IgE levels rise and then start falling and are expected to return to normal levels within 1–2 months after the beginning of the severe exacerbation [69]. When compared to other biomarkers of airway eosinophilia (e.g., blood eosinophils and FeNO), serum IgE seems to be a poor predictor of asthma exacerbations [70,71]. However, free serum IgE is reduced in response to omalizumab, and this reduction is associated with fewer exacerbations [72]. Consequently, increased levels of total serum IgE are a good biomarker for the screening of patients that will respond to omalizumab before treatment initiation [73,74].

Eosinophil-derived neurotoxin (EDN), is released by eosinophils, and, when found in serum, it may be a marker of eosinophil activation. It has been reported that EDN levels decrease after treatment with anti-IL5 agents (e.g., benralizumab), meaning that EDN could also be used as a response biomarker for these biological agents [64]. EDN has shown a similar sensitivity in studies [75,76] when compared to serum eosinophils, suggesting that EDN levels could also indicate the extent of eosinophilic airway inflammation [77].

It is well known that eosinophil peroxidase (EPO) is released from eosinophils following stimulation by an IgE-dependent mechanism [78]. In the study of Sanz et al., the EPO serum levels were higher in severe asthmatic patients when compared to those in healthy controls. It is thus suggested that EPO be used as an eosinophilic activation biomarker in asthma for the early discrimination between eosinophilic and non-eosinophilic asthma [79]. In this study, the EPO levels were correlated with the peripheral blood eosinophil count as a reflection of blood eosinophilia. On the contrary, Durham et al., found that EPO significantly decreased in asthmatic patients in comparison with high levels of other granular secretions, such as EDN [80].

Concerning periostin, which is referred to as osteoblast-specific factor 2, is a matricellular protein that mediates cell activation and promotes subepithelial fibrosis. Periostin can be secreted by bronchial epithelial cells and subepithelial fibroblasts as a response to mediators such as IL-4 and IL-13 [81,82]. Following secretion, periostin enters the bloodstream and therefore can be easily measured in serum; it is suggested as a systemic biomarker of airway eosinophilia [82,83]. However, the sensitivity of periostin was proven to be inferior when compared to blood eosinophils and FeNO [70]. Furthermore, IL-13-induced periostin upregulation established serum periostin as a probable biomarker of the response to anti-IL13 agents (lebrikizumab, tralokinumab) during clinical trials [84,85], although later on, serum periostin levels were considered to be not specific for severe asthma inflammation [86].

### 7.2. Sputum Eosinophils

The sputum eosinophil count obliquely reflects the eosinophilic airway inflammation levels and is therefore a sensitive and specific noninvasive diagnostic biomarker. The procedure encompasses either spontaneous or induced sputum collection from the individuals [87,88]. The data from numerous clinical trials indicate that a cell count of > 2–3% is considered diagnostic of eosinophilic airway inflammation [89]. The vast majority of severe asthmatics with high levels of sputum eosinophilia will respond to corticosteroids and targeted biological anti-eosinophilic therapies. Specifically, anti-IL-5 (mepolizumab, reslizumab), anti-IL-5 receptor α (benralizumab), and prostaglandin D2 receptor antagonist (fevipiprant) reduce sputum eosinophilia, and the efficacy of IL-4 receptor (dupilumab) has already been proven [90,91,92,93]. What is questionable is the efficacy of the biological agents targeting IL-13 (tralokinumab, lebrikizumab) [94,95].

The diagnostic, monitoring, responsive, and predictive value of sputum eosinophilia as a biomarker is unfortunately attenuated by the complex and time-consuming process of sputum induction and quantification. Point-of-care alternative methods, such as EPO in upper airway swabs, are therefore under development due to these challenges [96].

### 7.3. Fractional Exhaled Nitric Oxide (FeNO)

As a biomarker indicating eosinophilic airway inflammation, FeNO was initially suggested by the American Thorasic Society (ATS). The 2011 ATS guidelines recommended that low FeNO (<25 ppb) correlates with the absence of severe eosinophilic asthma, while high FeNO (>50 ppb) is diagnostic for eosinophilic airway inflammation. GINA guidelines advise that increased FeNO indicates residual Th2 inflammation for patients under treatment [97]. FeNO generally correlates with blood eosinophilia in most cases but not always, as FeNO and eosinophilia derive from different Th2 inflammatory pathways. According the 2020 ATS/ERS guidelines, it seems that it would be very useful to combine FeNO and eosinophil measurements in clinical practice in order to better guide the management of patients with uncontrolled, severe asthma. GINA recommends FeNO as a predictive biomarker of the available biological agents as well. High levels of baseline FeNO come with an adequate response to omalizumab, lebrikizumab, and dupilumab, while these levels are suppressed after treatment initiation. Raised FeNO levels could also predict the prognosis of severe asthmatics. Elevated FeNO at baseline was correlated with a bad prognosis and accelerated lung function decline in difficult-to-treat asthma, even in patients with normal spirometry at baseline [98]. Furthermore, persistent high levels of FeNO could be used as an indication of non-adherence to ICS treatment in clinical practice. In a small clinical trial, a rapid fall in FeNO was noted after 7 days of directly observed ICS (DOICS) treatment in non-adherent patients with “difficult- to-treat asthma”, and that decrease in FeNO was significantly greater than that in adherent patients. Thus, FeNO could be a useful tool to monitor adherence to ICS [99].

### 7.4. Exhaled Breath Condensate (EBC)

The evaluation of EBC is another noninvasive diagnostic technique used in severe asthma assessment. Exhaled Breath Condensate (EBC) is a biofluid directly obtained from the airway lining fluid non-invasively. The compounds being quantified in the EBC are mainly cysteinyl leukotrienes, which have been related to frequent exacerbations [100]. The pH of the EBC is also evaluated and seems to be reduced in asthma exacerbations, whereas low lipotoxin A4 in EBC is associated with severe asthma and declining lung function. Mediators of oxidative stress such as hydrogen peroxide H_2_O_2_ and 8-isoprostane were increased in steroid-naïve patients compared to the control [101]. Recently, there is increasing interest in conducting metabolomic analysis in EBC. The ATS/ERS recommendations on the EBC sample collection procedure and the technical standards of EBC analysis were published in 2017 and should serve as a guide for future studies, as it seems to be a promising material for obtaining a better understanding of asthma pathology and management [101].

### 7.5. Urinary Biomarkers

The urinary metabolite repertoire changes significantly during asthma exacerbations, and the content shifts to increased levels of alkanes and aldehydes. EPO secreted by eosinophils promotes the generation of brominated products in response to oxidative stress. Particularly, high bromotyrosine levels in urine were associated with uncontrolled asthma and an increased risk of exacerbations [102]. However, concordance with other severe asthma biomarkers (sputum eosinophils, FeNO) is not adequate [103]. According to studies, bromotyrosine could also be used as a biomarker of the response to steroid treatment, as its concentration in urine has been found to be decreased during steroid treatment [104].

### 7.6. OMICS

Regarding transcriptomics, Kuo et al., created a different approach to inflammatory endotyping using asthmatics from the U-BIOPRED cohort. According to this approach, three transcriptome-associated clusters (TACs) were identified. The first is TAC1, with the highest enrichment of gene signatures for IL-13/Th2 and innate lymphoid cell type 2 (ILC2) associated with the highest sputum eosinophilia. This grouped patients with severe asthma with oral corticosteroid dependency, frequent exacerbations, and severe airflow obstruction. The second is TAC2, and the third is TAC3. TAC2 and TAC3 are not associated with Th2 inflammation. TAC2 is characterized by elevated INFγ, ΤΝF-α, and inflammasome-associated genes, whereas TAC3 indicates metabolic and mitochondrial clusters [19,105,106].

Baines et al., investigated the gene expression profiles in the induced sputum specimen of asthmatic patients [107]. The study revealed six gene expression markers: alkaline phosphatase, Charcot–Leyden crystal protein (CLC), carboxypeptidase A3 (CPA3), chemokine receptor 2 (CXCR2), tissue-nonspecific isozyme (ALPL), and deoxyribonuclease l-like 3 (DNASElL3). The expression of this gene panel is correlated with a better response to corticosteroids and could help in distinguishing asthma endotypes [108]. Ongoing proteomics research in airway tissues identified a great variety of compounds that are elevated in severe asthmatics (e.g., IFN-γ, PDGFBB, IL-2, TNF-β, CCL27, CXCL7, CTAP-III, HPLN1, trypsin2, cathepsin G, ARSB, etc.) when compared to Th2-low asthma. Novel anti-inflammatory monoclonal antibodies targeting these molecules could be produced in the future [108,109].

### 7.7. Micro RNAs

Over the last decade, a variety of microRNAs have been linked to different diseases with profound Th2 activity. For instance, miR-21, miR-135a, miR-142, miR-143, miR-146b, miR-193b, miR-223, miR-365, miR-375, miR-452, and miR-1165-3p are only some of them [110]. Mi-RNAs profiling could interestingly contribute to distinguishing clinically inactive asthma from completely healthy individuals or predicting which patients will favorably respond to the available therapies [111].

A very promising application of the miRNA breakthrough is in differentiating asthma from chronic obstructive pulmonary disease (COPD). In everyday clinical practice, the discrimination between the two situations is very challenging, as they share a lot of clinical features. This is particularly difficult in the case of chronic severe asthma, where airway remodeling is observed. According to research studies, molecules such as miRNA-338 and miRNA-145 in sputum analyses could be utilized to distinguish patients with severe eosinophilic asthma from COPD patients [112].

## 8. Biologic Agents Targeting Type 2 Inflammation

**Figure 2:**
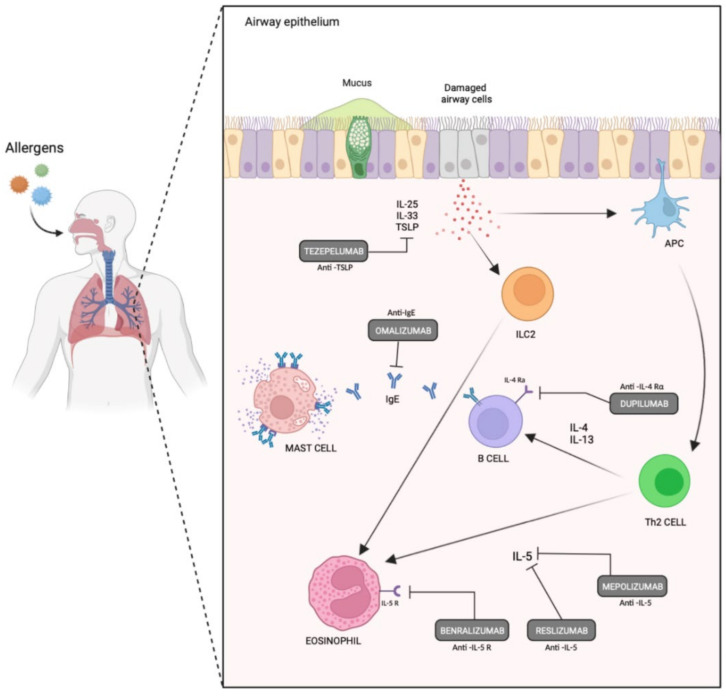
Currently used biologic agents and their sites of action targeting Th-2 inflammation in severe asthmatics.

In recent years, the development of biologic agents targeting the chain of pathogenic events leading to Th2 inflammation at different levels has significantly changed severe asthma management on a global level. Current guidelines on the management of severe asthma suggest that the patient’s inflammatory phenotype should be assessed and that an add-on Th2-targeted biologic should be considered for eligible patients. The currently used biologic agents and biomarkers appear in Table 3, and the biologic agents’ sites of action appear in Figure 2.

IgE is the primary immunoglobulin involved in Th2-high inflammation, so an anti-IgE antibody (omalizumab) was the first to be developed and has been approved for patients ≥ 6 years of age with moderate-to-severe allergic asthma. Omalizumab binds to the third constant region of IgE, preventing its binding to the FcεRI receptor, which is expressed primarily on basophils and mast cells. The eligibility criteria for this biologic include poor asthma control on conventional therapy, sensitization to inhaled allergen(s) on skin prick testing or specific IgE, increased total serum IgE, a body weight within the local dosing range, and more than a specified number of exacerbations within the past year [19,27,30,105]. The results from numerous randomized clinical trials (RCTs) and real-life studies have demonstrated that omalizumab has a good safety profile, improves asthma control, lung function, and quality of life, and reduces exacerbations, emergency visits, hospitalizations, and the use of oral corticosteroids [19,27,30,105,113]. In addition, the evidence also suggests that omalizumab is more effective in patients with higher levels of Th2 inflammation biomarkers, such as peripheral eosinophil levels [113]. However, the presence of autoantibodies and immune complexes in allergic airways could impede the action of omalizumab [113].

In addition, the major role of IL-5 in the differentiation, maturation, and survival of eosinophils led to development of agents that target IL-5. Mepolizumab and reslizumab are antibodies that bind to IL-5, preventing eosinophil activation. The combination of blood eosinophil counts and FeNO levels are considered as useful predictors of exacerbations in mepolizumab-treated patients. Another antibody, benralizumab, blocks the IL-5Rα, inhibiting the effect of IL-5 and resulting in eosinophil apoptosis through antibody-dependent cell-mediated cytotoxicity (ADCC) [105,113]. Eligible patients present with more than a specified number of severe exacerbations in the last year and an elevated blood eosinophil level (e.g., ≥150 or ≥300/μL). Data from RCTs and real-time observational studies show that these agents improve asthma control, lung function, and quality of life, while reducing severe exacerbations and blood eosinophils. Benralizumab has found to deplete peripheral blood basophils [114]. Oral corticosteroid use was also reduced with mepolizumab or benralizumab in comparison with placebo [19,27,30,105,113,114,115,116,117]. Despite their comparable effects, anti-IL-5 and anti-IL-5Rs have different mechanisms of action, and their effects vary depending on specific asthma endotypes. Some eligible patients may show suboptimal responses with anti-Il-5 biologics. This could be attributed to the presence of innate immune deficiencies, an alternate autoimmune pathology, or even inadequate doses of biologics [19].

Several clinical trials were conducted to develop biologics that target IL-4 and/or IL-13. Two anti-IL-13 agents, lebrikizumab and tralokinumab, were extensively studied, but the clinical outcomes were not satisfactory. An anti-IL-4 biologic, pascolizumab, was also used in studies, with disappointing results [27]. While the isolated blockade of either IL-4 or IL-13 has not been shown to be effective in severe asthma, the dual blockade of IL-4 and IL-13 has been promising [19,27,113]. Dupilumab, a biologic that inhibits both IL-13 and IL-4 by binding to the α-subunit of the IL-4 receptor, has been demonstrated to significantly decrease exacerbation rates and corticosteroid use and to improve symptom control and lung function [19,27,105,113,116]. Dupilumab is indicated for the treatment of patients with more than a specified number of severe exacerbations in the last year and increased Th2 biomarkers (e.g., blood eosinophils ≥ 300/μL or FeNO ≥ 25 ppb) or who require the use of oral corticosteroids. [11] Anti-IL-4/IL-13 agents mainly reduce airway hyperreactivity (AHR), showing sub-optimal results in patients with AHR and airway inflammation [19].

Currently, several novel biologic agents are under investigation, including inhibitors of the thymic stromal lymphopoietin (TSLP), anti-IL-33, and anti-IL-25. Alarmins TSLP, IL-33, and IL-25 are released by bronchial epithelial cells upon contact with pathogens, promote the production of Th2 cytokines, and result in Th2-high inflammation [19,30,113]. Tezepelumab, an anti-TSLP antibody which regulates Th2 immunity through Th2 and ILC2 cells, has been shown to reduce severe asthma exacerbations, blood eosinophil levels, total serum IgE, and FeNO and to improve FEV1 [19,30,105,113,114]. It is the only biologic approved by the FDA for severe asthma with no phenotype (e.g., eosinophilic or allergic) or biomarker limitations. Another anti-TSLP antibody (CSJ117) is currently used in clinical trials. An anti-IL-33 antibody, REGN3500, and the IL-25 blockade have been shown to prevent airway remodeling and AHR in animals, but studies on humans are awaited [113].

## 9. Future Perspectives on Treatment

The wide availability of monoclonal antibodies for T2-high asthma allows the physicians to have a more personalized approach regarding the selection of the most appropriate agent for each patient. However, the challenge is that almost one-third of the patients exhibit overlapping allergic and eosinophilic phenotypes [118] and are eligible for more than one treatment option. In the case of an inadequate response to the initial treatment, there is now the possibility to switch to another agent. Real-life data of switches from omalizumab to mepolizumab or benralizumab have started to be published with satisfactory results [118,119]. After 12 months of treatment with mepolizumab and benralizumab, asthma exacerbations and blood eosinophils were reduced, and pre-bronchodilator FEV1 as well as the asthma control test score (ACT) were improved [118,119]. There are also emerging real-world data on the switch from mepolizumab or reslizumab to benralizumab, with favorable outcomes in a considerable proportion of patients [120]. Dupilumab has also been proven to be an effective option for switches from either anti-IgE or anti-IL5/5Ra, also leading to a decrease in oral corticosteroids [121] A 4-month trial is the minimum needed to assess a patient’s response to therapy according to the GINA consensus for the management of severe asthma, and the decision for the switch has to be made by the treating physician without a predefined algorithm (GINA 2019).

A novel therapeutic approach, such as a combination of anti-IL-5 biologics with agents targeting other Th2 pathways, is studied. Until now, limited data exist for the use of combination biologics upon the treatment of severe persistent asthma. Case reports have been published referring to the combination of biologic therapies with controversial results [122,123]. The eligible patients were subjects that, despite clinical improvement on one biologic agent during the first 6 months of treatment, still did not meet the goals of therapy and have too high of a risk to discontinue the initial agent before achieving steady-state concentrations of an alternate drug [123]. Physicians consider de-escalating to a single agent after 3 to 6 months of combination treatment. For this purpose, further investigation on the precise inflammatory profiles that would benefit the most in combination with other biologics is needed.

## 10. Conclusions

During the last two decades, the development of biologics is promising in the management of severe asthma. Despite that fact, a significant burden of severe uncontrolled asthma remains. This may be due to the wide clinical heterogeneity of asthma phenotypes and endotypes or the lack of data on distinct factors that could predict a suboptimal response to therapy or guide the treatment option among available agents. Although there is great progress in identifying and managing endotypes with T2-high inflammation, T2-low asthma remains a challenging and not-deeply-understood endotype with limited therapeutic options. Tezepelumab and other agents under investigation will probably contribute to the management of this challenging endotype, but the pathophysiology should be further explored.

Furthermore, the implementation of personalized therapy is not widely applicable in clinical practice. The key to personalized medicine is the understanding of the immunology of asthma and the detection of biomarkers with high predictive and prognostic value for all different endotypes. Research should focus on biomarkers linked to the treatment choice and the prediction of clinical failure and exacerbations. Clinicians should approach every patient with difficult-to-treat, severe asthma based on the updated GINA guidelines, focus on the precise characterization of phenotypes and endotypes, and regularly review the response to targeted therapy.

## Figures and Tables

**Table 1:** Different types of biomarkers and their clinical application.

Types of Biomarkers	Clinical Application
Diagnostic	Confirm the presence of a disease or medical condition
Monitoring	Assess the presence, status, or extent of a medical condition
Response	Evaluate the response to a clinical intervention
Predictive	Identify patients more likely to experience an effect (positive or negative) after the exposure to a medical product or an environmental agent
Prognostic	Identify the likelihood of a clinical event, disease recurrence, or progression in patients with a medical condition
Safety	Predict toxic adverse events induced by drugs, medical interventions, or environmental agents’ exposure
Risk	Indicate the potential for developing a disease or medical condition in an individual not currently presenting a clinically apparent medical condition

**Table 2:** Biomarkers of severe eosinophilic asthma and their clinical implication.

Biomarkers	Biological Sample	Clinical Implication
Eosinophils	Blood/Sputum	Indicative of airway eosinophilia Response Predictive Monitoring
IgE	Blood	Predictive Response
EDN	Blood	Indicative of airway inflammation Predictive Response
Periostin	Blood	Predictive Response
EPO	Blood	Indicative of airway inflammation
Neutrophils	Sputum	Indicative of airway inflammation
FeNO	Exhaled breath	Indicative of airway eosinophilia Monitoring Response Predictive
EBC	Exhaled breath	Response
Bromotyrosine	Urine	Predictive Response
Omics: ALP, ALPL, CLC, CPA3, CXCR2, DNASElL3	Blood	Indicative of airway inflammation Response Predictive
Micro-RNAs: miR-21, miR-135a, miR-142, miR-143, miR-146b, miR-193b and miR-223, miR-365, miR-375, miR-452, miR-1165-3p	Blood	Indicative of airway inflammation Predictive Response

EDN: eosinophil-derived neurotoxin, EPO: eosinophil peroxidase, EBC: exhaled breath condensate.

**Table 3:** Phase III and IV biological agents targeting Th-2 inflammation and relevant suggested biomarkers indicative of a response.

Biological Agent	Target	Route of Administration	Relevant Biomarkers (Response/Predictive)
Omalizumab	IgE	SC	Sputum Eosinophils FeNO IgE
Mepolizumab	IL-5	SC	Blood/Sputum Eosinophils
Reslizumab	Il-5	IV	Blood/Sputum Eosinophils
Benralizumab	IL-5 receptor α	SC	Blood/Sputum Eosinophils EDN
Dupilumab	IL-4 receptor	SC	FeNO
Tezepelumab	thymic stromal lymphopoietin	SC	*

EDN: eosinophil-derived neurotoxin, SC: subcutaneous, IV: intravenous. * no established biomarker for this biologic agent.

## Data Availability

Not applicable.
